# Dental treatments reform for older adults in Israel: the first six years

**DOI:** 10.1186/s13584-026-00767-y

**Published:** 2026-06-05

**Authors:** Ayelet Berg-Warman, Shlomo Paul Zusman, Ile Kermel Schifman, Shai Sade, Lena Natapov

**Affiliations:** 1 Myers-JDC-Brookdale Institute, Jerusalem, Israel; 2 University of Rochester, Rochester, NY USA; 3 Ariel University, Ariel, Israel; 4 Ministry of Health, Jerusalem, Israel

**Keywords:** Dental care for aged, Health Care Organizations, Health Care Reform

## Abstract

**Background:**

In 2019, a Universal Health Coverage reform of dental services for older adults was implemented in Israel, in order to improve their access to dental care, reduce financial obstacles and decrease inequalities in service utilization. This study aims to examine changes in dental care among elderly over the first 6 years of the reform.

**Methods:**

Analysis of the utilization reports submitted by the HMOs to the Ministry of Health between the years 2019 (the beginning of the reform) to 2024. The reports included number of patients treated by type of treatments each year.

**Results:**

During the first six years, the number of treated patients in the framework of the reform increased from 46.6 thousand to 129.4 thousand. During the years 2019 to 2024 there was a significant increase in preventive and first aid treatments (2.7 and 2.2-fold, respectively). Maintenance and surgical treatments increased moderately (1.8 and 1.6-fold, respectively),as well as rehabilitative treatments for 80 + age group and check-ups (1.5 and 1.4-fold, respectively).

**Conclusions:**

During the first six years of the dental treatment reform, there was a notable increase of older adults who received dental treatments at HMOs clinics.

## Plain Language Summary

Prior to 2019, dental care for older adults was paid mainly out-of-pocket. In 2019, dental services for them were included in the basket of medical services that the residents of Israel are entitled to under the National Health Insurance Law. As part of this reform, the four Health Maintenance Organizations (HMOs) are obliged to provide these services. We analyzed the HMOs reports submitted to the Ministry of Health during the years 2019–2024. The reports included numbers of older adults who received various types of treatment each year.

In the first 6 years of the reform there was a significant increase of almost threefold in the number of patients treated in the HMOs’ clinics. The most significant increase was in preventive and first aid treatments. Nevertheless, there is still a long way to go to reach optimal uptake of these dental services.

## Introduction

The state of oral and dental health has a significant impact on the quality of life and nutrition of older adults. Oral health of older adults in Israel is comparatively poorer than in many of the developed countries [1]. Part of the reasons involves barriers, such as the high cost of treatment and a lack of awareness regarding the importance, of oral health which hinder older adults in Israel from obtaining dental services [2].

In the “Mabat Zahav” national survey, it was found that quality of life of 17% of older adults in Israel is detrimentally affected by their oral health [3]. In another study which used the national survey data, significant differences were found in the consumption of calories, protein and nutritional fiber, a situation partly explained by oral and dental health status. Thus, among older adults who reported problems in chewing due to their dental condition there was a lower consumption of these nutritional components. It was also found that the consumption of a variety of nutrients by elderly individuals with dentures was lower than among those with natural teeth (21 natural teeth and 4.1 pairs of teeth on average) [4].

An examination of the oral health of older adults in Israel, as measured according to the rate of edentulousness and the number of teeth the individual has, indicates that in 2020 24% of older adults over the age of 65 had lost their teeth while the rest had 19 teeth on average. Among the 75–84 age group, there were 20 teeth and among the 85 + group there were only 13 [5]. According to the WHO, an individual needs 20 teeth for normal functioning [6]. In a study published in 2001 [7], it was found that 70% of the low-income elderly in Israel had lost all of their teeth, as compared to 45% of the high-income elderly (about one-third of all households with the highest income earners) [2]. Recent data still reveal pronounced socioeconomic disparities: 39% of older adults with financial difficulties were edentulous, compared with only 19% among those without such difficulties [5]. 

The data on the consumption of dental services by older adults indicate that in 2020 18% of the 65 + age group received dental treatment in an HMO clinic while 40% visited a dentist for a routine checkup [5]. This represents an increase relative to the situation 22 years earlier, when only 21% of the 65 + age group had routine dental checkups [7]. It was also found that 53% of the 65 + group visited a dentist during the past year, 28% of whom went for a routine checkup (35% among individuals with a high socioeconomic status and 12% among those with a low socioeconomic status) [8]. In the study by Berg-Warman et al. [9], it was found that the most common reason for not visiting a dentist when there was a need to do so was the cost of treatment. According to the Social Survey for 2013 by Central Bureau of Statistics [CBS] [10], 43% of the respondents who needed dental treatment decidided not to get it. In a study to evaluate the “Smile Again” project, which was meant to increase the accessibility of dental services to low-income individuals by means of mobile clinics, it was found that 65% of patients did not complete their dental treatment due to the cost and 88% felt that the cost of dental treatment is too high in general. About 51% decided not to get treatment in the first place due to its cost [11].

In past studies, emphasis was placed on the importance of including preventative, restorative and reconstructive dental care within the basket of services according to the National Health Insurance Law (NHIL) [2, 7].

“The National Health Insurance Law (NHIL) was enacted in 1994 and providing Universal Health Coverage (UHC) set the state’s responsibility for health care provision to all citizens. Health care is provided by four Health Maintenance Organizations (HMO). Dental care was not included in the basket of health care services, except oral and maxillo-facial surgery in cases of trauma and tumors, and limited care to special groups such as oncology patients” [1].

“Until 2010… dental care was paid for primarily out of pocket, constituting about 10% of national health expenditure, higher than in many Western countries. Yet, most industrialized countries have better dental health. It was a market failure; the high costs did not translate into a better oral health for Israeli citizens” [12].

In 2010 UHC was expanded and dental care for children was included into the National health Insurance Law (NHIL). At the start of the reform, the entitlement age was from birth to 8-years. The eligibility age was increased gradually and, in 2019, reached 18-years.

In 2019, a reform of dental services for older adults was introduced. Its goal is to improve access to dental services for older adults and to mitigate some of the barriers. As part of the reform, routine care for the 75 + age group was included in the basket of services under the NHIL, and in October 2019 rehabilitative care for the 80 + age group was also included. Since July 2022, the entitlement was expanded, and all citizens aged 72 + are eligible for preventative, restorative and prosthetic care. However, in 2020 43% of the 65 + age group and only 28% of the 85 + age group were aware that dental treatment for older adults was now part of the HMOs’ health basket [9]. Another study conducted in 2024 among adults aged 70 + found that only 36% were aware of the reform [13].

The State Comptroller of Israel report (May 2023) indicates significant gaps in the utilization of dental care entitlements within the Health Maintenance Organizations, particularly among older adults (aged 75+), with low uptake rates (sometimes below 15% in certain regions). The report found that lack of public awareness of the reform, along with insufficient dissemination of information by the health funds, are the primary contributing factors. Fewer than one-third (29%) of older adults were aware of their eligibility for free or subsidized dental care, and 61% reported lack of awareness of the reform as the main reason for non-utilization [14].

The goal of the research was to examine the changes in dental care utilization over the first six years of the reform, i.e. including the dental services for older adults in the basket of services of National Health Insurance Law.

## Materials and methods

Analysis of the HMOs’ activity in the realms of the NHIL reports submitted to the Ministry of Health between the years 2019 (the beginning of the reform) and 2024. The reports included number of patients treated by type of treatments each year.

## Results

**Fig. 1:**
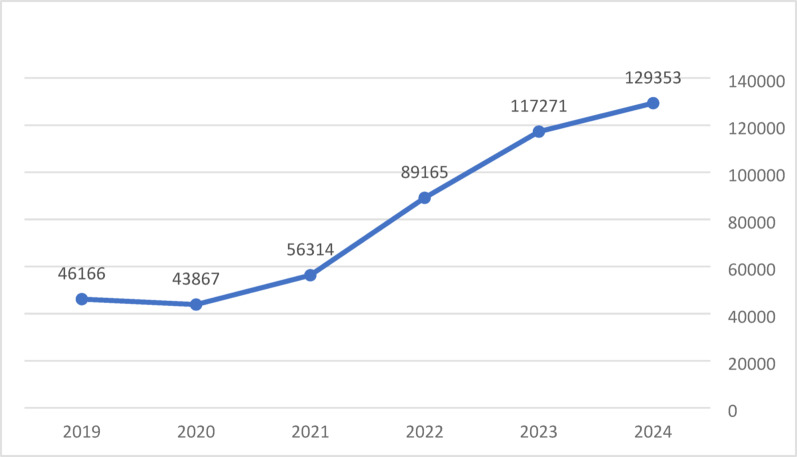
Number of members who received treatment by year

According to the HMOs reports submitted to the Ministry of Health, 46,166 members (who constitute 10.7% of all members aged 75+) received some kind of treatment in 2019; in 2020, the number was 43,867; and in 2021 it had growth to 56, 314 (who constitute 11.8% of all members aged 75+). In September 2022 the reform was expanded to 72 + age group. As a results in 2024 the number of members aged 72 + who received dental care increased to 129,353 (who constitute 17.1% of all members aged 72+) (Fig. 1).

Table 1 presents the proportion of patients among members aged 75 + of all the HMOs during the years 2019–2024. It can be seen that the most common treatments were preventative and maintenance checkups. It also shows that between 2019 and 2024, there was an increase in the proportion of patients who received preventive treatment – from 4.7% to 12.8% (increased by 2.7-folds); those who received first aid – from 1.1% to 2.5% (increased by 2.2-folds). The increase in maintenance treatment and surgical treatment was more moderate. Those who had a surgical treatment increased from 1.8% to 3.2% and dental checkup – from 6.5% to 6.9%. It also appears that there was an increase in the proportion of members aged 80 + who received rehabilitative treatment between 2020 (after the implementation of the reform) and 2024 (from 4.5% to 6.9%).

**Table 1:** The percentage of patients treated within the age group, by type of treatment^

Year	First Aid	Dental Checkup	Prevention	Maintenance	Surgical	Rehabilitative (for 80+)
2019	1.1	4.8	6.9	2.7	1.8	
2020	1.4	4.7	5.4	2.6	1.9	4.5
2021	2.5	7.1	7.5	2.9	2.2	5.3
2022	2.8	8.2	9.0	4.3	2.6	6.2
2023	2.3	6.5	11.2	4.6	3.1	7.1
2024	2.5	6.9	12.8	4.3	3.2	6.9
2024/2019	2.2	1.4	2.7	1.6	1.8	1.5^^

^ Years 2019–2022 include aged 75+, Years 2023–2024 include aged 72+ ^^ 2024/2020

## Discussion

The goal of the research is to examine the pattern of dental care utilization by the elderly over the first six years (2019–2024) of the implementation of dental care reform, which included the dental treatments for older adults under the National Health Insurance Law.

In preparation for the reform, staff dentists underwent training in the treatment of older adults and efforts were made to publicize the reform among this population [14]. Initial efforts to advance elderly oral health reforms were undertaken, including awareness campaigns and outreach initiatives. These efforts involved publicizing the reform across various media sites and channels by both the Ministry of Health (MOH) and the HMOs, alongside communication activities carried out by the medical and administrative staff in the clinics. It should be also mentioned that according to a study carried out among older adults in early 2020, most of the 75 + age group were not aware of the dental health reform [9, 13]. No additional clinics dedicated to the reform were established by the HMOs prior to the anticipated rise in the number of patients.

An analysis of the data on the utilization of dental services shows a significant increase in the proportion of patients who received treatment as part of the reform between 2019 and 2024 – from 46,166 (10.7% of insured individuals among 75 + age group) to 129,353 (17.1% of insured individuals among 72 + age group). The most significant increase was in the percentage of older adults who received preventive treatments (a rise of 2.7-fold) and first aid (2.2-fold). The increase in the proportion of older adults who received preventive treatment is encouraging since this is the most beneficial type of dental treatment. The World Health Organization (WHO) emphasizes the importance of developing programs aimed at disease control, prevention, and intervention among vulnerable populations, including older adults [15]. Furthermore, it is a starting stage for more complicated rehabilitative treatment.

Evidence suggests that intervention programs incorporating dental treatment can effectively prevent infections and illnesses such as influenza, pneumonia, and fever [16]. Additional initiatives have focused on increasing referrals from general practitioners to dentists [17], providing educational campaigns, implementing preventive and screening measures, and subsidizing dental care for economically disadvantaged older adults [18].

Systematic oversight and quality monitoring of dental services for older adults were limited prior to 2025. In 2025 the Ministry of Health has initiated formal quality control and inspections in HMO dental clinics, following the allocation of dedicated supervisory positions. These inspections can provide more detailed insights into service provision, accessibility, the integration of routine oral care into general health services offered by HMOs, and gaps in care, enabling refinement of policy recommendations and supporting the effective implementation of the reform.

A more moderate increase occurred in the proportion of patients who received surgical treatment (1.8-fold) and maintenance treatments (1.6-fold). There was an increase in the percentage of patients who received rehabilitative treatments (1.5-fold), following their inclusion in the basket provided by the HMOs. Rehabilitative treatment is essential for elder adults who require it. It is expected that as more elderly finish the preparatory stages, the reparatory treatment will increase too.

Although the proportion of older adults receiving treatment under the reform has increased, it still remains lower than the rate observed among children in their reform in the 2010s. The study to evaluate the reform in dental health among children that was carried out in 2013 and a report by the Ministry of Health indicate that 33%–45% of children aged 2–12 utilized their eligibility to receive service at the HMO clinics [19]. According to Jarallah et al. [20], between 2011 and 2020 the rate of usage by children increased from 18.3% to 35.4% (there are no data on the use of dental services for the years preceding the implementation of the reform). This apparently provides evidence for the gradual increase in awareness among the public in the context of a reform.

Despite the significant increase in the percentage of dental services utilized by older adults during the first six years of the reform, the percentage is still very low compared to that of children. It is important to recall that during the COVID-19 pandemic there was a recommendation to defer non-emergency dental treatment for older adults [21] and after the pandemic the demand for treatment increased significantly. However, the increase was smaller that the one seen in the children’s reform. In any case, there is a need to continue monitoring the changes in the consumption of services and the adjustments made by the HMOs for a longer period.

The MOH, together with the Gertner Institute for Epidemiology and Health Policy Research, has recently initiated the development of a new tool for measuring waiting times to assess accessibility of public dental services. This monitoring system, scheduled for fased implementation for children, is planned to be extended to elderly populations in the future, which will help identify barriers and improve service delivery. Furthermore, the first set of quality indicators for dental services provided by HMOs has already been introduced for children. These indicators will enable systematic tracking of care coverage and quality and are expected to be expanded in the future to include older adults. It is anticipated that the ongoing supervision of HMOs will further support the integration of oral health with general health services, enhancing preventive care, multidisciplinary referrals, and overall health outcomes for older adults. Together, these initiatives are expected to strengthen monitoring, accountability, and the quality of care provided to the elderly population.

## Limitations of the study

In most cases, the data in dental services research describes only those who accessed care, leaving the status of those who did not unknown.

The study was conducted during the COVID-19 pandemic, during which movement restrictions and lockdowns were imposed in Israel to cope with the outbreak. During this period, strict measures were taken to protect the health of older adults, especially in 2020. They were asked to remain in their homes and avoid visiting clinics for dental treatments. Even after clinics resumed their activities, older adults were reluctant to attend due to fear of infection. This was reflected in a decline in the number of patients and treatments, which limited the ability to examine the impact of the reform on the consumption of dental services and made it difficult to draw conclusions about the reform’s initial effects.

In view of the available data there is no possibility to determine the ratio between professional treatment needs and the treatments that were actually provided.

Another limitation is related to the data provided to the research team by the HMOs; only some of the data from the HMOs were quarterly, while others were annual, which allowed only the presentation of annual rather than quarterly data. In other words, trends could be examined over only six points in time.

## Conclusions

Prior to the implementation of the reform, the preparations of the HMOs to provide dental services to older adults primarily focused on the training of professional staff. There were no significant changes to existing infrastructure or adding infrastructure to provide dental treatment to this population. In retrospect, although there was a significant increase in the proportion of older adults receiving dental treatment during the first six years of the reform, the percentage of utilization remains low.

## Policy recommendations for improving oral health care for older adults

1. Strengthening National Oversight and Information Dissemination: The Ministry of Health should mandate and actively supervise systematic efforts by Health Maintenance Organizations (HMOs) to inform older adults about their statutory entitlements under the dental care reform. These efforts should emphasize the established contribution of regular oral health care to overall health, functional capacity, and quality of life in older age.
2. Targeted and Evidence-Based Outreach by HMOs: HMOs should be required to develop and implement targeted, evidence-based outreach strategies aimed at increasing awareness and understanding of available preventive and restorative dental services among eligible older members. Communication should be clear, accessible, and tailored to the needs of older populations, explicitly detailing eligibility criteria and pathways to care.
3. Integration of Oral Health into Routine Geriatric Care: Oral health assessment and promotion should be formally integrated into routine health services for older adults. HMOs should ensure structured training of healthcare professionals—including physicians, nurses, and nutritionists—to recognize oral health needs, provide basic guidance, and initiate timely referrals to dental services as an integral component of comprehensive geriatric care.
4. Implementation of Oral Health Quality Indicators: HMOs should be required to develop, adopt, and routinely report standardized oral health quality indicators specific to the elderly population. These indicators should include, at minimum, service timeliness, accessibility, patient-reported outcomes and satisfaction, coverage of preventive care, and outcomes of rehabilitative treatments, in order to enable continuous quality improvement.
5. Systematic Monitoring of Accessibility and Utilization: HMOs should systematically measure and publicly report key dimensions of accessibility to dental services, including geographic coverage, provider availability, waiting times, and utilization rates among eligible older adults. Identified gaps—particularly those affecting peripheral or socioeconomically disadvantaged populations—should be addressed through targeted corrective actions.
6. Regulatory Oversight and Accountability: The Ministry of Health should assume a proactive regulatory role in overseeing the implementation of the dental care reform for older adults. This oversight should ensure adherence to quality standards, equitable access, effective patient engagement, and accountability across HMOs, with enforcement mechanisms where deficiencies are identified.

## Data Availability

The datasets used and/or analysed during the current study are available from the corresponding author on reasonable request.
